# Health Professionals’ Approaches to Support Patient Diversity in the Assessment of Vaginismus: A Critical Feminist Qualitative Study for Inclusive Care

**DOI:** 10.3390/healthcare14101261

**Published:** 2026-05-07

**Authors:** Rashmi Pithavadian, Vijayasarathi Ramanathan, Sowbhagya Micheal, Tinashe Dune

**Affiliations:** 1 School of Health Sciences, Western Sydney University, Locked Bag 1797, Penrith, NSW 2750, Australia; 2 Faculty of Medicine & Health, University of Sydney, 3 Parramatta Rd, Camperdown, NSW 2050, Australia; vijay.ramanathan@sydney.edu.au; 3 Translational Health Research Institute, Western Sydney University, Locked Bag 1797, Penrith, NSW 2750, Australia; s.micheal@westernsydney.edu.au (S.M.); tinashe.dune@cdu.edu.au (T.D.); 4 School of Medicine, Western Sydney University, Locked Bag 1797, Penrith, NSW 2750, Australia; 5 Faculty of Health, Charles Darwin University, Ellengowan Drive, Darwin, NT 0909, Australia

**Keywords:** vaginismus, genito-pelvic pain/penetration disorder, sexuality, well-being, LGBTQIA+ health, age and disability, intersectionality, critical feminism

## Abstract

**Background/Objectives:** Current research on vaginismus predominantly represents White cisgendered and heterosexual women of reproductive age. It is unclear how health professionals (HPs) navigate and support the needs of patients with vaginismus who are gender, sexually, ethnically, religiously, age and/or disability diverse. Therefore, this qualitative study explored health professionals’ experiences and perceptions of patient diversity to holistically assess and support people with vaginismus. **Methods:** In 2023–2024, 23 HPs in general practice, uro/gynaecology, pelvic floor physiotherapy, mental health, nursing and clinical education participated in semi-structured interviews. Data were inductively thematically analysed with a critical feminist poststructuralist focus on heteronormativity, cisnormativity, ethnocentricity, chrononormativity, and able-bodied normativity. **Results:** Two themes were developed. The first theme on ‘uneven attention of diversity dimensions in the assessment and support of vaginismus’ explored patients’ ethnicity, religion, sexuality, gender, age and disability. The second theme on ‘sexually and gender-diverse people’s varied treatment goals for vaginismus’ examined nuanced challenges between heterosexual and non-heterosexual women and limited representation of gender-diverse people. **Conclusions:** The findings suggest that not discussing patients’ diversity may contribute to their identity erasure and ethnocentric exaltation of White centrality. Treatment approaches may uphold heteronormativity. HPs often described vaginismus as a young woman’s problem. It is recommended that HPs review whether patients with advancing age and/or disability suppress desires for pain-free sex due to societal norms. Decolonising approaches and abject theory could inform the development of inclusive health resources. This can assist HPs to sensitively and supportively assess patients’ diversity to improve their holistic health and well-being outcomes for vaginismus.

## 1. Introduction

It is often difficult to gain appropriate healthcare assessment and support for vaginismus [1]. Vaginismus can make any type of vaginal penetration, including sexual intercourse, tampon insertion, or gynaecological examination, painful, difficult and/or impossible, depending on severity [1,2]. Effective treatment for vaginismus involves a multidisciplinary approach of psychotherapy, biomedicine, and pelvic floor therapy administered by different health professionals (HPs) [3]. However, accessing appropriate assessment and support from multidisciplinary HPs for vaginismus is challenging. The lack of social awareness of vaginismus and stigma of sexual health hinders women’s ability to recognise when and where to seek and receive help for their symptoms [1]. This is compounded by the limited knowledge of vaginismus and its management among HPs in healthcare contexts [4]. Such factors contribute to people’s experiences of misdiagnosis, improper differential diagnosis and unsuitable treatment for vaginismus, including endometriosis-related surgery and hymenotomy [1,5]. This can prolong and exacerbate the negative impacts of vaginismus on people’s relationship consummation and intimacy, family planning, sexual expression, and mental health, including anxiety, depression, and self-esteem [3,6,7]. In these ways, people with vaginismus who do not receive appropriate healthcare can experience compromised mental health, holistic well-being, and quality of life.

Current research on healthcare assessment and support for vaginismus and its associated challenges is focused on White cisgendered and heterosexual women [1,8]. People’s ethnic or religious cultural backgrounds can influence their health literacy, health communication, and perceptions and reservations towards discussing sexual health problems [7,9]. Emerging scholarship reports on ethnically and religiously diverse women’s healthcare-seeking experiences for painful sex from the patient perspective [9,10,11,12]. This still leaves a paucity on HPs’ perspectives to navigate and support the needs of ethnically and religiously diverse people with vaginismus in healthcare practice. There is also lacking research on healthcare assessment and support of sexually and gender-diverse people with vaginismus [1]. Moreover, people have varied social positions and identities, which can change over time. These can shape their intersections of diversity dimensions of gender, sexual orientation, ethnicity, religion, age and disability [13,14]. This has effects on their health presentation and accessibility to healthcare [14,15]. It must be examined in healthcare practice for vaginismus to gain insights for HPs to inclusively support wider demographics of people with the condition.

Current structural critiques of the healthcare system focus on patriarchal or androcentric inclinations [10,16,17,18]. This has uncovered how women’s complaints of painful sex have been normalised as a part of womanhood, and/or dismissed in healthcare practice by androcentric myths of female fragility such as women’s lowered pain tolerance [5,18,19]. However, the healthcare system in Western countries has also been found to reproduce ideologies of heteronormativity, cisnormativity, ethnocentricity, chrononormativity and able-bodied normativity [20,21,22,23]. This refers to how the healthcare system is structured to privilege quality of care to those who identify as heterosexual; cisgender (one of the binary genders assigned to their sex at birth); Westernised in culture and religion; young; and able-bodied without disability [23,24]. The implications of this on healthcare for diverse people with vaginismus have not been explored.

Healthcare assessment and support for diverse people with vaginismus can be nuanced compared to other causes of painful sex or dyspareunia such as vulvodynia, endometriosis, or gynaecologic cancers [5]. Symptoms of vaginismus, including triggers, duration, and location of pain, and treatment for it can be similar but vary from other conditions that cause painful sex [5]. Therefore, vaginismus is the focus of this study rather than the broader category of genito-pelvic pain/penetration disorders (GPPPDs) which amalgamated vaginismus and dyspareunia in the Diagnostic and Statistical Manual (DSM) 5 [25]. This is relevant for clinical practice and research where vaginismus as a diagnostic category is still used [3,26,27]. No research has explored how HPs factor diversity dimensions into their healthcare practice in patients’ presentations of vaginismus. Such research can generate findings to inclusively support the mental and holistic well-being of those with marginalised or intersecting experiences of the condition, particularly for healthcare systems with multicultural or diverse populations. Therefore, this qualitative study sought to address the following research question: What are health professionals’ experiences with and perceptions of patient diversity to holistically assess and support people with vaginismus?

## 2. Methods

### 2.1. Theoretical Frame

This study is informed by a critical feminist poststructuralist (CFPS) lens which does not claim positivist neutrality. Instead of viewing the healthcare system as inherently neutral, poststructuralism examines the possible power structures that contribute to the disempowerment and healthcare inequity of certain groups [28]. A critical feminist approach orients poststructuralism to examine how structures beyond the patriarchy can marginalise women and other minorities [29]. The authors recognise that sexism continues to pose significant barriers for the majority of women’s gynaecological healthcare irrespective of background; however, this study examines the implications of other often-overlooked structural factors.

People’s identities as heterosexual, cisgender, White, young, and able-bodied are socially perceived as the default or norm in Western societies [30]. This socially constructs heteronormativity, cisnormativity, ethnocentricity, able-bodied normativity and chrononormativity (to follow linear and standardised life stages as part of ageing, including the expectation for losing interest in sex as part of women’s ageing) into normative and hegemonic structures across societal systems including the healthcare system [30,31,32,33]. These normative structures have been overlooked in research on vaginismus. Therefore, the effect of these structures will inform this study. Such normative structures marginalise those who deviate from them and can lead to structural bias and prejudice against others through heterosexism, cisgenderism, racism, ageism, and ableism [28,30].

The CFPS lens examines how the healthcare system’s upholding of normative structures marginalises diverse people with vaginismus in healthcare assessment [29,34]. Therefore, this study’s CFPS lens will examine how HPs’ perspectives reveal the ways that patients’ diversity is considered in healthcare practice for vaginismus under the healthcare system’s normative structures. This can illuminate how normative structures affect HPs’ ability to enact their individual care goals to assess and holistically support people with vaginismus [30,35].

### 2.2. Study Design

A qualitative study design was employed to capture HPs’ perspectives without imposing close-ended questions [36]. This allowed rich exploratory and unconsidered findings on an under-researched aspect of healthcare assessment and support for diverse people with vaginismus [36]. The standards of reporting qualitative research (SRQR) were followed [37]. This study is part of a larger research project that received ethics approval by the Human Research Ethics Committee at Western Sydney University on 21 August 2023 (Approval Number: H15587).

### 2.3. Semi-Structured Interview Guide

Semi-structured interviews both oriented participants to reflect on patients’ diversity in their healthcare practice while allowing them to drive discussion to unanticipated topics. The semi-structured interview guide questions that form this study’s basis are presented in Table 1. These questions were extracted from the Supplementary File of the semi-structured interview guide for the larger project, which was developed following Kallio et al.’s framework [38]. This involved reviewing the literature and expert opinion to develop interview guide questions which were field and pilot tested [38]. Given the academic terminology of CFPS concepts of heteronormativity, cisnormativity, ethnocentricity, able-bodied normativity/ableism and chrononormativity/ageism, these terms were avoided in the development of the interview guide questions. The interview guide questions were phrased with simpler conversational words focused on diversity.

### 2.4. Procedure

To be eligible to participate in this study’s interviews, HPs had to have consulted at least one person with vaginismus and practice in Australia. Using purposive sampling between September 2023 and January 2024, the authors circulated the larger project’s outline, participant eligibility criteria, AUD 50 voucher incentive, and contact information to various health organisations and their professional networks. This included the Society for Australian Sexologists (SAS); Westmead Fertility Centre; Nepean Blue Mountains Local Health District and Primary Health Network; Women’s Health Centres in Bankstown, Liverpool, and Blue Mountains; Benevolent Society Campbelltown; and networks of HPs across Australia including 289 GPs. Snowballing also occurred between HPs for recruitment. In total, with snowballing, 34 HPs expressed their interest to participate. The first author screened whether they met the eligibility criteria of: being a health professional, having consulted at least one patient with vaginismus, and practicing in Australia.

Twenty-three eligible HPs then reviewed the participant information sheet and provided consent to participate. The first author, who has postgraduate qualifications and a professional background in qualitative research, conducted and audio-recorded all interviews via Zoom Workplace videoconferencing software (institutional version provided and regularly updated by Western Sydney University) between October 2023 and February 2024. The interviews averaged 68 min (range 47–99 min). After each interview, the first author recorded research memos of her initial thoughts, reflections or interpretations, in an online document shared with all authors [39]. The audio-recorded interviews were transcribed using Trint web-based transcription software (its continuously updated version was accessed during 2024) with a naturalistic style retaining idiosyncrasies of speech. Transcripts were de-identified with participants being assigned pseudonyms. The first author undertook transcript verification by playing all audio recordings against transcripts to make corrections, and simultaneously added further research memos. These transcripts were emailed to participants for member-checking, which 21/23 of them completed [40].

### 2.5. Data Analysis

For inductive thematic analysis, the first author read all transcripts twice for data immersion [41], and reflexively reviewed her research memos. This informed her development of preliminary parent and child codes that were labelled to align with CFPS perspectives to answer the research question and indicate how HPs viewed diversity dimensions of gender, sexuality, ethnicity, religion, age and disability to assess and support people with vaginismus [41]. There was a larger focus on certain diversity dimensions in the analytic coding due to the distribution of the interview data and participants’ less detailed responses on other diversity dimensions. All authors reviewed and revised the preliminary codes’ labels [41]. The first author then used Quirkos 2.5.3 software as a tool to undertake line-by-line coding of the transcripts according to the preliminary codes [42]. The first and last authors met four times to review coding on Quirkos to merge, delete, and rename code labels [41]. Both authors shared their reasoning for any disagreements in coding until they either reached a consensus or presented it to the other authors to provide their perspective until majority agreement was attained. Data adequacy was reached when no new codes could be generated to answer the research question within the study’s scope [43]. This left two preliminary parent and corresponding child codes, which were finalised as two themes and underlying subthemes organised to answer the research question and uphold CFPS theory. These were reviewed by all authors and changes were discussed until consensus was reached for inter-coder consistency [44].

### 2.6. Researcher Reflexivity

The authors have varied professional backgrounds as clinicians and/or researchers in sexual health, biopsychosocial health, and health sociology [45]. This informed the study’s analytical strength to focus on the multifaceted and holistic dimensions of patient assessment and support for vaginismus. The authors’ own diverse gender, ethnic, religious, age, and sexual health profiles supported a critical and balanced review of the study’s findings to minimise focalising certain dimensions of diversity. However, dimensions of diversity outside of the authors’ backgrounds which could be relevant to healthcare for vaginismus may have been overlooked [45].

### 2.7. Qualitative Rigour

The study’s qualitative rigour and trustworthiness were upheld in the following ways [46,47]. For study transferability, researcher reflexivity and the theoretical frame of the CPFS lens are outlined. The number of participants that informed each theme is stated with participants’ quotes to demonstrate that the findings are data-driven for confirmability. Transcripts were spot checked by the first author and member checked for credibility. The semi-structured interview guide, theoretical frame, and methodological process for data collection and analysis are provided to support study replication as part of dependability.

## 3. Results

The sample of participants included 23 HPs in general practice, uro/gynaecology, pelvic floor physiotherapy, mental health, nursing and clinical education. Their full demographic information is presented in Table 2. The analysed data from interviews are presented below as two themes and corresponding subthemes. The number of HPs who reported consultations with patients by diversity type is reported in Table 3. Participants’ quotes include their pseudonym, HP type, sex (F or M), years of professional experience (y.exp), age range, region type, and geographical states of their practice in New South Wales (NSW), South Australia (SA), Victoria (VIC), or Queensland (QLD) in Australia.

**Theme 1: Uneven attention of diversity dimensions in assessment and support of vaginismus**

***1.1: Narrow consideration of sexual orientation and overlooked gender diversity in presentation formulation***

When asked about their patients’ diversity beyond ethnicity and religion, several HPs stated that nothing was raised in consultations. When prompted on sexual diversity among patients, 15 HPs mentioned that they had one or more patients with vaginismus who did not identify as heterosexual. While seven of these HPs referred to their non-heterosexual patients as being lesbian or bisexual, others did not know or state their patients’ sexual orientation. Only some HPs moved beyond noting patients’ sexual orientation to holistically considering it in healthcare assessment. Such HPs commented that the “same-sex relationships that I come across will have pain because of some form of penetration. The only thing that changes that a little bit is trying to work out what… they’re using for penetration. Because some people do have, can get kind of allergic and skin reactions to some, products, like some toys and things… kind of a latex reaction to some things and it gives them a real burning vaginal sensation” and then “you might recommend changing to something like glass or metal and seeing if they have the same issue. Because you might actually identify that it’s a toy allergy as opposed to, you know, just straight vaginismus” (Tim, gynaecologist, M, 5 y.exp, 35–45 y.o., rural, VIC). These HPs discerned patients’ sexual orientation to inform their process of history-taking and differential diagnosis. Such HPs reviewed patients’ sexual orientation to collect sexual history on their types of vaginal penetrative activities, such as the use of sex toys, condoms, or lubricants, to differentiate between allergies versus vaginismus.

Some HPs conflated sexual and gender diversity. When probed, eight HPs noted gender diversity in their patients with vaginismus. While five of them mentioned having gender non-binary patients with vaginismus, two HPs reported consulting patients with vaginismus who were transgender. They stated “someone identifies as non-binary. Yeah, where there’s that gender diversity then, and that’s probably something that I don’t actually know of any physiotherapists that specifically kind of identifies that or say that they are friendly in that area” (Felicity, GP, F, 6 y.exp, 25–35 y.o., metropolitan, NSW). These HPs recognised a gap to support gender-diverse patients with vaginismus. They did not explicate how patients’ gender diversity informed their healthcare assessment of vaginismus.

***1.2: Ethnic and religious diversity observed but often not negotiated in healthcare management***

All participants commented on the varied diversity of patients with vaginismus with mixed findings. Eight HPs had a majority or all Caucasian patients with vaginismus. Several noted Asian, Indian subcontinental and Middle Eastern heritages among patients. Two participants reported that “nearly zero people of African descent or Black people” (Victoria, uro/gyno, F, 10 y.exp, 35–45 y.o., metropolitan, SA and VIC) presented with vaginismus which also appeared “less so in the Polynesian or Pacific Island population” (Xena, uro/gyno, F, 10 y.exp, 35–45 y.o., metropolitan, VIC). One participant shared that “in the last probably 2 or 3 years, there’s been a probably substantial increase in the sense of Aboriginal and Torres Strait Islander women” with vaginismus (Ben, GP, M, 27 y.exp, 35–55 y.o., metropolitan, NSW).

Fifteen HPs noted that their patients with vaginismus had religious backgrounds. These religions were largely Muslim and Christian, with Catholic, Hindu, Buddhist and Sikh backgrounds as the next most frequent. HPs discussed that patients’ conservative cultural and religious backgrounds contributed to reservations towards sexual health and help-seeking for it. Some viewed this to be more prevalent in non-Anglo/European patients. Other HPs observed that “when you go back through the family legacies, there’s cultural issues that come in. They present as sort of Anglo-Saxon Australian women, but cultural burdens are absolutely part of the picture” (Ariella, psychosexual therapist and clinical sexuality educator, F, 3.5 y.exp, 45–55 y.o., regional, QLD). These HPs did not overlook how traditional Western upbringings or strong Christian backgrounds among Caucasian or Anglo-European women shaped their reservations towards vaginismus. While only two HPs outlined asking specific questions on the influence of patients’ religious or cultural background on their presentation, another participant mentioned avoiding any discussion of it with patients.

Seven HPs moved beyond identifying patients’ diversity to sharing their strategies to support them. A HP shared that:

“I had a Coptic patient that, I worked with their priest to help her to accept that it was religiously and culturally okay for her to do the homework exercises, which, you know, were inserting her own dilator and touching her own genitals” (Megan, GP/mental health, F, 40/13 y.exp, 65+ y.o, metropolitan, NSW).

These HPs shared their strategies, such as involving religious figures or making referrals to similarly diverse HPs, to respectfully negotiate patients’ diverse cultural and/or religious beliefs that conflicted with treatment rationales. This supported patients’ engagement in treatment.

***1.3: Age-based assumptions and restricted focus on disability in screening and examination***

Ten HPs viewed help-seeking to resolve vaginismus symptoms as a younger women’s problem, associated with more active sexual lives, higher sexual expectations, or family planning goals during reproductive ages. They felt that this was not the case for older women: “it’s probably more like a generational thing. They will, you know, tolerate the pain, right. I think it’s a generational, like they’re tougher, they’re more like ‘okay so pain it is’” (Louisa, F, GP, 13 y.exp, 45–55 y.o., metropolitan, NSW). HPs discussed that older women tended to endure through their vaginismus symptoms rather than treating it or stopped help-seeking after they “ruled out anything sinister” (Denise, GP, F, 5 y.exp, 35–45 y.o., metro and regional, NSW and SA). Some of these HPs self-referenced the need to assess further in this demographic: “And older, again, it’s the post-menopausal women that I’m just not asking, but I’ll change that” (Denise, GP, F, 5 y.exp, 35–45 y.o., metropolitan and regional, NSW and SA). They reflected on how vaginismus being viewed as a younger women’s problem means that older women were not screened for it, which they planned to change in their own practice.

When asked about relevant co-/multi-morbidities and diversity among patients with vaginismus, mixed findings were reported among HPs. Five HPs explicitly mentioned disabilities of neurodivergence; cognitive impairments; physical disabilities, including spina bifida and cerebral palsy; and mental health disabilities including trauma. Two HPs discussed the challenges to gain informed consent from patients with cognitive disabilities to undertake physical examination. Another HP explained how she altered her approach for physical examination in patients with a physical disability: “if they physically are limited from a disability standpoint that I cannot do an adequate procedure, then we’ll do an examination under anaesthesia” (Victoria, uro/gyno, F, 10 y.exp, 35–45 y.o., metropolitan, SA and VIC). Only three HPs discussed how they factored patients’ disability when assessing for vaginismus in terms of how to support their needs for physical examination.

**Theme 2: Sexually and gender-diverse people’s varied treatment goals for vaginismus**

***2.1: Nuanced challenges in treatment goals of heterosexual versus non-heterosexual women***

HPs were asked if there was a difference between heterosexual and non-heterosexual women’s help-seeking goals for vaginismus. Most responded with long contemplative pauses or stated that they did not consider that or notice a difference. A HP commented that “the goals are often, by the time they get to me, never been outlined” (Victoria, uro/gyno, F, 10 y.exp, 35–45 y.o, metropolitan, SA and VIC). Many HPs perceived that heterosexual women’s goals to seek help for vaginismus was to satisfy male partners’ needs or have children, particularly if there was cultural or religious pressure. Some heterosexual women’s goals were reportedly to achieve pain-free or even pleasurable penis-in-vagina (PIV) sex. Eleven HPs explained that non-heterosexual women had different treatment goals compared to heterosexual women with vaginismus. These HPs shared:

“Their [lesbian women’s] goals were related to being able to experience more internal pleasure. I find lesbians are really bloody good at outercourse, *way* [emphasis] better than heterosexual women in terms of their knowledge base and their-, so it’s usually more as an add on-, like to be able to experience the additional pleasure of internal stimulation” (Sarah, physio, F, 30 y.exp, 45–55 y.o., regional, SA).

Such HPs discussed that many lesbian women’s goals were to improve their vaginismus symptoms to expand and enhance their sexual pleasure from outercourse to intercourse with vaginal penetration. HPs highlighted that “for lesbian women, GPPPD/vaginismus does not seem to have such a relationship ending impact as it can do for some heterosexual women” (Rachel, physio, F, 18 y.exp, 45–55 y.o., metropolitan, SA) and “people who are not heterosexual probably have more options in terms of how they can work with their partner to mitigate the pain” (Sydney, GP, 9 y.exp, 25–35 y.o., metropolitan and regional, NSW). These comments indicated HPs’ perspectives that vaginismus caused less pressure in non-heterosexual women, and their treatment goals were often to improve their sexual quality of life. Three HPs shared that patients who were lesbians tended to receive more support from partners compared to heterosexual women, with one HP noting multiple cases wherein both same-sex partners had vaginismus.

Other HPs reflected on lesbian women whose treatment goals were not to achieve intercourse but to undertake self-swabs, pap smears, or gynaecological exams for routine health maintenance. These goals mirrored women with pelvic pain co- or multi-morbidities.

***2.2: Limited representation of gender-diverse people’s treatment goals***

Only some HPs discussed the gender diversity of their patients in relation to their treatment goals for vaginismus. One HP stated that “I only have one patient that I can remember who identified as non-binary and no, her, sorry, their treatment goal did not differ from the others. And I think it was more, you know, to make sex not painful” (Felicity, GP, F, 6 y.exp, 25–35 y.o., metropolitan, NSW). Other HPs noted that regarding “the main differences, I’ve noticed less relationship stress in the non-binary group” (Xena, uro/gyno, F, 10 y.exp, 35–45 y.o., metropolitan, VIC). Commonalities across these HPs’ statements highlighted that their gender-diverse patients had treatment goals which centred on improving vaginismus symptoms to become sexually active through forms of vaginal penetration. However, there were emerging comments that gender-diverse patients might experience less relationship distress in having vaginismus compared to cisgendered women.

## 4. Discussion and Directions for Future Research and Practice

This qualitative study captured 23 health professionals’ (HPs) recounted experiences and perspectives of patients’ diversity to help assess and holistically support presentations of vaginismus. In line with CFPS theory, the findings have been analysed in relation to the selected normative structures of heteronormativity, cisnormativity, ethnocentricity, chrononormativity and able-bodied normativity in the healthcare system. Given the sample size, the presented findings can be interpreted as exploratory and offering directions for future research and practice development in line with acceptable qualitative practice.

While participants were not unwilling to examine patients’ gender and sexual diversity, the results reported in Themes 1.1 and 2.1 indicated that it was largely not raised nor considered in consultations. This may suggest that heteronormativity and cisnormativity were possibly reproduced by HPs, often unconsciously, through the educational curricula and training they received to assess patients’ presentations [48]. The reported lack of focus on diversity can render deviations of GSD as marginalised and invisible through a CFPS lens, which risks making gender and sexually diverse (GSD) people feel psychosocially excluded even in healthcare contexts [49]. GSD patients’ unique challenges and needs are thus often not visible and overlooked in the healthcare system’s formal assessment processes among HPs [49]. This can be problematic given that transmasculine people have a higher incidence of dyspareunia, which can be psychosocially detrimental [50]. A few participants’ reported conflation of gender and sexual diversity suggests a lack of differentiation due to heteronormativity and cisnormativity being default in the healthcare system.

While scholarship describes vaginismus as negatively impactful for heterosexual women’s relationships with men and family planning [3], this study’s data found that non-heterosexual women with vaginismus experience different psychosocial challenges not focused on penis-in-vagina (PIV) sex. As noted by this study’s participants, GSD people’s treatment goals may involve achieving more pleasurable sexual outercourse or vaginal insertion with fingers or sex toys. However, patients’ treatment goals are not assessed beyond PIV sex in existing psychometric measures for vaginismus, including the vaginal penetration cognition questionnaire (VPCQ), multidimensional vaginal penetration disorder questionnaire (MVPDQ), Vaginal Penetration Skills Scale (VPSS), and vaginismus subscale in the Golombok Rust Inventory of Sexual Satisfaction (GRISS) [51,52,53,54]. Additionally, while there are emerging clinical guides for GSD patients with non-pain-related sexual dysfunctions [55], this is lacking for GSD people with genito-pelvic pain conditions such as vaginismus. There is only minimal assessment information and one sentence on treatment for GPPPDs in the Clinical Management Guidelines for Obstetrician–Gynecologists [56]. Future research could develop psychometrically validated tools and clinical guidelines or frameworks to guide HPs to sensitively determine patients’ possible gender and sexual diversity and how it shapes their treatment goals. The development of such healthcare resources should strive for non-intrusiveness that avoids heteronormative and cisnormative presumptions to inform holistic assessment. This could help to give visibility to the treatment goals and needs of otherwise overlooked and marginalised GSD identities in those with vaginismus to more inclusively support their healthcare management and psychosocial well-being.

The results of participants’ observed contemplative pauses and comments that they did not consider or notice differences in non-heterosexual patients’ treatment goals suggest the heteronormativity of vaginismus treatment modalities [1]. Completion and success of progressive physical therapy with graduated vaginal trainers is often defined by readiness for PIV sex, which is a heteronormative endpoint reflected in practices including the use of male surrogate partners for patients without participating partners [57,58]. Such treatment could be more inclusively adapted to view success and completion not by reaching the largest trainer size or PIV sex [7]. Rather, completion may benefit in being reframed to whichever vaginal trainer size aligns with the patients’ goal for insertion, such as digital insertion or vaginal examination, as reported by participants. This can potentially support patients’ goals for sexual agency to use fingers or sex toys in sexual activity and/or healthcare agency to use tampons or undertake gynaecological examinations [59].

The study participants’ emerging comments on lesbian couples both having vaginismus suggest a need for further examination in future studies. It mirrors research on heterosexual women with vaginismus having male partners with sexual dysfunction [27,60,61]. The participants’ comments on lesbian partners being more supportive may be explained by scant scholarship on queer women with dyspareunia having higher sexual communication, understanding of their partner’s dyspareunia and orgasm attainment due to their similar anatomy, and access to non-heteronormative sexual scripts [62,63]. Consequently, participants’ perspectives that vaginismus is less straining on relationships could be explored in larger empirical studies. This can provide a clearer evidence-based understanding of lesbian couple’s experiences of vaginismus to better support their needs in healthcare management. Nonetheless, this study’s exploratory findings suggest that determining same-sex or gender-diverse partners’ relevant sexual health presentations and level of support can inform more inclusive assessment steps for HPs. This can help guide HPs to modify existing heteronormative partner homework exercises or couples counselling for vaginismus to suit GSD couples and uphold more holistic care that aligns with their treatment goals [57].

Participants’ recounts of patients, who were not heterosexual or had co-/multi-morbid pelvic pain conditions, seeking treatment for non-penile vaginal insertion, pap smears, self-swabs, or gynaecological examination call for a review of current multidisciplinary treatment approaches for vaginismus. The conventional multidisciplinary combination of pelvic floor exercises, graduated insertion of vaginal trainers (dilators), and psychotherapy focuses on treating patients’ negative mental cognitions and physiological difficulty associated with penile penetration [64,65,66]. Such approaches in multidisciplinary treatment could be reframed to accommodate and normalise other types of vaginal penetration in clinical practice and research. More inclusive acronyms, such as penetrative vaginal sex (PVS) or vaginal penetrative activity (VPA) for asexual people or those who seek non-sexual vaginal penetration, may be useful as alternatives to decentre PIV sex and to reframe discourse [67]. Such discourse may help to restructure the healthcare system to extend focus beyond PIV sex to inclusively consider GSD people’s psychosocial experiences of vaginal penetration difficulties. This can potentially direct attention to under-examined psychological and physical therapy for intersex people, or even transgender people with neovaginas, who may contract vaginismus [68].

Participants reported reservations to enquire about patients’ cultural, ethnic and religious background for holistic healthcare management may reflect Western ethnocentricity [69]. Their reservations might suggest lacking confidence or knowledge, or considerate avoidance of unintended offence. Regardless of the reason, the commonality can also reflect their training and educative discourse [35,70]. Such training and education may uphold a colourblind racial approach which aims for neutrality [71]. However, patients often seek diversity-sensitive care [72]. This is particularly relevant for effective health outcomes in vaginismus given that studies have reported the effects of religiosity, ethnicity, and sociocultural factors in patients’ vaginismus aetiology and therapy responses [73,74,75]. People’s different cultural, ethnic and religious upbringing or beliefs can also affect their health literacy, language proficiency, health communication, ability to access services, and perceived discrimination in the healthcare system [7,76,77,78]. This can consequently influence patients’ level of engagement with healthcare management and treatment adherence for vaginismus [77,78]. Participants’ lacking discussion of patients’ non-Western cultural backgrounds in assessment may ethnocentrically homogenise Western culture as patients’ centrality and default in healthcare practice for vaginismus through a CFPS lens [1,79]. It is not feasible to expect a lengthy discussion on patients’ religious values to inform treatment. Rather, clinical practice frameworks with steps, precise wording, or phrases may help to address HPs’ reservations. It can guide HPs to comfortably and respectfully assess patients’ cultural, ethnic or religious diversity that is relevant to inform and amend their healthcare management plan for vaginismus [80].

Participants’ comments on low patient presentation from Black or Indigenous backgrounds may warrant further exploration. There is increased reported medical pain and distrust towards Western healthcare systems among Black and Indigenous populations [81,82,83]. Barriers amongst Aboriginal and Torres Strait Islander peoples seeking healthcare include racism, shame, and geographical distance [82]. Black women’s descriptions of vulvovaginal pain often do not align with traditional categorisations of genito-pelvic pain, which can contribute to delays to receive the correct assessment, diagnosis, and treatment of vaginismus [80,84,85]. These may be indicative of a residual continuation of Eurocentric colonial agendas to erase Black and Indigenous peoples’ experiences [86,87]. Such insights suggest the utility of decolonising approaches to dismantle long-established colonial agendas covertly embedded in healthcare practice at systemic and organisational levels [87]. Addressing this at the macro levels of clinical education and training can support systemic change to be realised at the individual practice levels to support the biopsychosocial needs of Black and Indigenous peoples with vaginismus [87].

The limited participants reporting that they moved beyond noting patients’ religious diversity to adapting it into tailored healthcare management strategies may reflect the otherwise biomedical secularity of the Western healthcare system [88]. It could potentially contribute to the diverse religious backgrounds of patients, including Westernised Christianity noted by participants, being overlooked in clinical assessment of vaginismus. This aligns with research suggesting that cultural competency in healthcare practice may be insufficient to identify and address relevant aspects of patients’ religious backgrounds to treat painful sex [89]. Such lacking cultural competency can be problematic given that patients’ religious upbringing includes abstinence idealisation, inadequate sex education, family and peer pressure, sexual rigidity, negative cognitions such as fear or shame towards sex, female genital cutting, and arranged marriage with pressured or lacking consent [9,12,90,91,92]. Therefore, it may aid HPs to respectfully negotiate any such religious factors to support patients’ navigation to engage in healthcare to improve their vaginismus [89,93]. This could include engaging faith-based figures whom patients trust, referring to social support services when appropriate, or reframing focus on sex-positive aspects of religion with patients to increase their engagement and motivation with treatment for vaginismus [7]. Such strategies could potentially facilitate healthcare practice to more comprehensively and holistically support ethnoculturally and religiously diverse people with vaginismus. This can support multicultural, secular, and multireligious countries to mitigate ethnocentrism.

When probed, some participants mentioned women’s experiences of having vaginismus with disability and/or post-menopause. These diversity dimensions, needing to be explicitly probed in interviews, suggest it could be ‘abject’, or cast aside and marginalised, because it disrupts ethnocentric or culturally constructed scripts or boundaries under a CFPS lens [94]. Vaginismus itself can be abject because the symptoms present a paradox of people consenting to vaginally penetrative sex yet it being involuntarily painful, difficult, and distressing. This contradicts cultural boundaries of consensual painless sex versus consensual painful and enjoyable sex in sadomasochism. Due to being disruptive, the abject is sequestered, marginalised, and made taboo [94]. This is to maintain the status quo in the healthcare system for most who align with culturally constructed boundaries at the expense of deviating minorities [95].

The study’s results suggest that vaginismus being viewed as a young person’s problem may inadvertently and covertly perpetuate ageism in healthcare practice by abjecting ageing women with vaginismus. Ethnocentric cultural scripts on sexuality are often patriarchal. They highly value neurotypically able-bodied and youthful reproductive women. The cultural script of chrononormativity positions post-/menopausal women as reaching a life stage of losing sexual desire alongside fertility [96,97]. Participants’ quoted willingness to change reflects that structural normativity may have unknowingly shaped their healthcare practice, which could therefore be reviewed and revised to support inclusive care for patients with vaginismus. While it is acknowledged that women’s ageing correlates with decreased sexual desire due to reducing oestrogen levels, HPs should strive to avoid over-generalising this trend to all older women and apply case-by-case assessment of individual goals.

Participants’ limited discussion of disability focused on consent, which is only one aspect of sexual and reproductive health and rights [98]. HPs may have not explicitly described endometriosis as a disability co-morbid with vaginismus because it does not neatly align with physical, neurodevelopmental, and cognitive disability scripts [99]. However, it may be clinically useful to assess co-/multi-morbid pelvic pain conditions as disabilities alongside vaginismus. This can help to tailor appropriate multidisciplinary treatment for complex presentation to achieve more holistic and effective health outcomes [100,101]. Able-bodied normativity scripts also culturally construct people with physical, neurodevelopmental, and cognitive disabilities as care dependent, non-agentic, and not able-bodied for reproduction or sex [102,103]. Thus, they may be abject from being prioritised in healthcare discussions related to sex for their vaginismus [94].

A participant’s reference to post-menopausal women who stopped help-seeking for vaginismus after ruling out sinister causes could be explored in future clinical practice and research. It can inform the development of specific validated history-taking questions and self-report measures to determine and respectfully probe on how patients’ diversity dimensions inform their psychosocial presentations [104]. The development of such healthcare tools would extend upon the current self-report measures used to multidimensionally assess sexual function and distress in vaginismus, including the female sexual function index (FSFI) and female sexual distress scale (FSDS), which do not review the role of ageing or disability in patients’ presentation of vaginismus [105,106]. Such tools may help HPs to determine and address when abjected or marginalised menopausal or disabled women still desire sex with vaginismus but do not seek help due to following ethnocentric cultural scripts of age and/or able-bodied normativity. Conversely, ageing and/or disabled people with vaginismus that clearly indicate that they do not desire sex should be respected and not disbelieved [107]. The CFPS application of abjection may be practically useful to identify and understand which groups are prioritised and psychosocially excluded in the healthcare system. It can inform targeted strategies, including revisions in HPs’ educational curricula, training, and policy to restructure and extend the Western healthcare system’s constructed boundaries to integrate marginalised groups and support more inclusive quality healthcare [98].

## 5. Limitations

The study focuses on normative structures in Western healthcare systems. The findings lacked perspectives from men and gender-diverse HPs because most participants were women. No additional funds were available for targeted recruitment of gender-diverse HPs. Apart from sexual, gender, religious, and ethnic diversity being explicitly asked in interviews, HPs were generally asked to report on any other dimensions of diversity. It is possible that HPs did not report on relevant experiences and perspectives of diversity dimensions among their patients with vaginismus that were not explicitly named.

## 6. Conclusions

This is the first study to explore health professionals’ (HPs) recounted experiences and perspectives to assess patients’ diversity to holistically support their presentations of vaginismus. A qualitative study design underpinned by critical feminist poststructuralism facilitated an examination of how the healthcare system’s normative structures of heteronormativity, cisnormativity, ethnocentricity, chrononormativity and able-bodied normativity affect diverse people with vaginismus in healthcare assessment. Gender and sexually diverse people tend to be overlooked in heteronormative treatment approaches for vaginismus that frame successful outcomes as PIV sex. The lack of discussion of patients’ diverse ethnic, cultural and religious backgrounds can risk ethnocentrically upholding White centrality, Black and Indigenous erasure, and secularity. This may marginalise relevant factors of patients’ ethnocultural and religious diversity which should be assessed to support patient engagement and treatment adherence. Chrononormativity and able-bodied normativity embedded in the healthcare system may lead to not determining whether ageing and/or disabled patients with vaginismus desire sex but might not seek support to follow societal norms. Decolonising approaches and abject theory have the potential to inform the development of practice guidelines and frameworks to support HPs to sensitively and supportively assess patients’ diversity to improve their psychosocial and well-being outcomes for vaginismus. This can be added to curricula, training and professional development to support HPs. It may enhance HPs’ skillsets to respectfully assess all patients on their treatment goals for vaginismus and know how to amend healthcare management accordingly to support tailored care for patients’ diversity. This can help to more holistically and inclusively support people with vaginismus in increasingly diverse, multicultural, and secular countries and societies.

## Figures and Tables

**Table 1:** Semi-structured interview questions focused on assessment and support of diverse patients with vaginismus.

Demographic questions	Did any of your patients with vaginismus identify as gender diverse such as non-binary gender or transgender? If yes, how many patients?
	Did any of your patients with vaginismus identify as lesbian, bisexual, pansexual, or non-heterosexual? If yes, how many patients?
	Did your patients with vaginismus indicate any ethnic diversity? If yes, what were their ethnic backgrounds?
	Did your patients with vaginismus indicate any other diversity? (probe co-/multi-morbidity, disability, age, etc.)
Semi-structured interview questions	How may patients’ diversity be a factor in treatment or healthcare management?
	What have patients’ treatment goals been? Probes *: Have treatment goals varied between heterosexual women versus those who were lesbian, bisexual, pansexual, or non-heterosexual? Have treatment goals varied between cis-gendered women versus patients who identified non-binary gender and/or transgender? Have treatment goals varied among patients from cultural or religious backgrounds? Have treatment goals varied between any other diverse groups? (e.g., age) * Ask only if HP indicated any patient diversity in responses to demographic questions
	How have patients’ help-seeking experiences influenced how they perceived themselves?
	Do you have any other comments to add that we have not already discussed?

Note: These are questions extracted from the interview guide of a larger project that informed this study’s findings.

**Table 2:** Participants’ demographic information.

**Health professional type** ^1^		**Health professionals’ self-identified gender**	
Pelvic physiotherapist	5	Cis/male	2
General practitioner	9	Cis/female	21
Uro/gynaecologist	5		
Obstetrician	1	**Health professionals’ self-identified ethnicity**	
Psychologist, therapist, counsellor	4	Anglo-/White/Caucasian/European-Australian	11
Gynaecology nurse	1	English	1
Sexuality educator	1	Russian	1
		Sri Lankan	1
**Years in practice**		Bangladeshi Australian	1
≤5 years	3	Chinese	1
6–10 years	5	Chinese Australian	1
11–15 years	7	Vietnamese Australian	1
16–20 years	1	Asian Australian	1
21–25 years	2	Iranian Australian	1
26–35 years	2	Ugandan	1
35+ years	3	Zimbabwean	1
		Afro-Canadian	1
**States health professionals practiced** ^1^			
NSW	11	**Health professionals’ age bracket (in years)**	
SA	7	25–35	5
VIC	6	35–45	7
QLD	1	45–55	7
		55–65	2
**Region type** ^1^		65+	2
Metropolitan	20		
Regional	6		
Rural	1		

^1^ Some participants had more than one answer.

**Table 3:** Number of health professionals (HPs) reporting consultations with patients by diversity type.

Patient Diversity Category	HPs (*n*)
**Sexual orientation diversity**	**15**
Lesbian	7
Bisexual ^1^	1
Unspecified (by HP) ^2^	8
**Gender diversity**	**8**
Non-binary	5
Transgender	2
Unspecified (by HP) ^2^	1
**Significant ethnic diversity**	**15**
**Limited ethnic diversity**	**6**
**No ethnic diversity** (all patients reported as White European)	**2**
**Religious diversity**	**15**
**Diverse age range**	**18**
**Limited age diversity** (predominantly younger, pre-menopausal patients)	**5**
**Disability**	**5**

^1^ One health professional reported consulting both lesbian and bisexual patients with vaginismus and was therefore counted in both subcategories. Therefore, subcategory totals do not sum to the overall total for sexual orientation diversity. ^2^ Unspecified indicates that the health professional reported consulting patients with sexual or gender diversity but did not specify the subtype.

## Data Availability

The dataset generated and/or analysed during the current study is not publicly available as per the conditions of the ethics approval. Data are located in mediated access data storage at https://doi.org/10.26183/s1rz-vx33. Please contact the corresponding author for information on the data.

## Supplementary Materials

The following supporting information can be downloaded at: https://www.mdpi.com/article/10.3390/healthcare14101261/s1, File S1: Interview guide from larger study.
