# Supplementary material for: Health Professionals’ Approaches to Support Patient Diversity in the Assessment of Vaginismus: A Critical Feminist Qualitative Study for Inclusive Care
Source: Healthcare (Basel). 2026 May 7;14(10):1261. doi: 10.3390/healthcare14101261 (PMC13205255; doi:10.3390/healthcare14101261)
Supplement: Supplementary file 1 [file healthcare-14-01261-s001.zip › healthcare-4210985-supplementary.pdf]

Supplementary File S1: Interview guide from larger study

Topic	Question
Demographic information	i. What type of health professional are you?
	ii. How many years have you been in practice?
	iii. What mode/s do you use to deliver your services to patients? (e.g., in-person, telehealth, or both)
	iv. How many patients with vaginismus have you seen in the past 6 months?
	v. How many patients with vaginismus have you seen in the last 5 years?
	vi. Did any of your patients with vaginismus identify as gender diverse, such as non-binary gender or transgender? If yes, how many patients?
	vii. Did any of your patients with vaginismus identify as lesbian, bisexual, pansexual, or non-heterosexual? If yes, how many patients?
	viii. Did your patients with vaginismus indicate any ethnic diversity? If yes, what were their ethnic backgrounds?
	ix. Did your patients with vaginismus indicate any other diversity? (probe religion, disability, age, etc.)
	x. Do you treat general health issues or specialise in women's health? If so, what aspect of women's health?
	xi. Which state and city, suburb, or town do you practice in? Have you always treated vaginismus in this area or anywhere else?
	xii. Would these areas be metropolitan, regional or rural?
	xiii. What qualifications did you receive to support patients with vaginismus? What years did you attain these qualifications? Did you attain any of these qualifications outside of Australia?
	xiv. What gender do you identify as?
	xv. What cultural or ethnic identity do you identify as?
	xvi. What is your age bracket (e.g., 25-35, 35-45, 45-55, 55-65, or 65+)? If you do not mind sharing, how old are you?
	xvii. How did you hear about this study?
History-taking	1. How did patients end up consulting you?<br>*Probes:*<br>- *What was their pathway to reach you?*<br>- *Were you the first point of contact?*
	2. What sort of information do you ask for when taking the patient's history?<br>*Probes:*<br>- *Does this include a psychosocial history?*<br>- *Do you take an initial or ongoing history?*
	3. Have patients mentioned consulting alternate health practitioners or tried complementary or alternative treatments?
	4. Have patients used self-help strategies (self-help kits with dilators, pelvic floor exercises, and mental health exercises)?
	5. How do you identify and respond to any challenges (e.g. trauma) that patients with vaginismus face to get better?

Assessment	6. How do you understand vaginismus in terms of what the condition is and the symptoms that you look for in patients?
	7. Did you learn about vaginismus during your degree or was it something you learned about through other qualifications or courses?
	8. How would you assess for vaginismus?<br>*Probes:*<br>- *Would you undertake an examination, use tools or questionnaires, or look for certain biopsychosocial factors in patients?*
	9. Under what circumstances would you not do a physical examination?
	10. What are your patients’ understanding of their genital anatomy?
	11. Had your patients heard of vaginismus before?
Clinical or diagnostic formulation	12. What is your understanding of different diagnostic categories for painful sex?
	13. How do you rule out other conditions for a differential diagnosis?<br>*Probes:*<br>- *How would you perform a differential diagnosis with vulvodynia?*
	14. Do you feel the need to diagnose vaginismus as primary or secondary and tell patients? Why or why not?
	15. Do you use the term genito-pelvic pain/penetration disorder (GPPPD) from the Diagnostic and Statistical Manual 5 (DSM-5)? Why or why not?
	16. Do you think dyspareunia should be merged with vaginismus for diagnosis? Why or why not?
	17. Do you use the term sexual pain-penetration disorder from the International Classification of Diseases 11 (ICD-11)? Why or why not?
Management plan and support	18. In what circumstances would you make referrals and what sort of health professionals or social support services do you refer patients to?
	19. What treatments do you prescribe patients and how do they respond to them?
	20. What may be challenges that patients experience with treatment and how do you address them?
	21. What have patients’ treatment goals been?<br>*Probes*:*<br>- *Have treatment goals varied between heterosexual women versus those who were lesbian, bisexual, pansexual, or non-heterosexual?*<br>- *Have treatment goals varied between cis-gendered women versus patients who identified non-binary gender and/or transgender?*<br>- *Have treatment goals varied among patients from cultural or religious backgrounds?*<br>*Ask only if HP indicated any patient diversity in responses to demographic questions
	22. If your patients have partners, do you involve them in management or treatment? Why or why not?
	23. How do you follow-up with your patients?
	24. How would you describe the level of awareness of female sexual pain disorders such as vaginismus and vulvodynia in healthcare training and

	general clinical settings compared to other sexual dysfunctions (probe, e.g., anorgasmia, female sexual arousal disorder, and male sexual dysfunctions)?
	25. How have patients' help-seeking experiences influenced how they perceived themselves?
Wrap up	Do you have any other comments to add that we have not already discussed?

Note 1: For consistency in the presentation of the interview guide, the term 'patient' is used. During interviews, the term 'client' was used when preferred by participants.

Note 2: Questions on the development of a resource output from the larger mixed-methods study will be reported in quantitative dissemination.
